# Immunocompromised Adults at Risk of Severe Varicella

**DOI:** 10.3390/reports9030222

**Published:** 2026-07-11

**Authors:** Satoko Minakawa, Toshihide Higashino, Daisuke Sawamura

**Affiliations:** 1 Department of Clinical Laboratory, Hirosaki University Hospital, 53 Honcho, Hirosaki 036-8563, Japan; 2 Department of Infection Control Center, Hirosaki University Hospital, 53 Honcho, Hirosaki 036-8563, Japan; 3 Department of Dermatology, Hirosaki University Graduate School of Medicine, 5 Zaifu-cho, Hirosaki 036-8562, Japan; smartdai@hirosaki-u.ac.jp; 4 Department of Dermatology, Self-Defense Forces Central Hospital, Tokyo 154-8532, Japan; toshihide-higashino@umin.ac.jp

**Keywords:** chickenpox, dermatomyositis, hepatic dysfunction, systemic lupus erythematosus, varicella-zoster virus

## Abstract

**Background and Clinical Significance**: Varicella, caused by the varicella-zoster virus (VZV), is typically a childhood disease; however, adult cases carry substantially higher morbidity and mortality. Immunocompromised individuals are particularly vulnerable to severe outcomes. Many adults in Japan lack documented varicella vaccination, representing a missed opportunity for preventing primary VZV infection. None of the patients had documented prior varicella vaccination. **Case Presentation**: We conducted a retrospective review of varicella cases at Hirosaki University Hospital between April 2007 and March 2025. Ten patients (eight adults and two children) were identified. Among the eight adult patients, written informed consent for publication was obtained from five patients, whose clinical courses are described in detail in this report. Four patients had underlying conditions requiring immunosuppressive therapy, and two were undergoing cancer treatment. All immunocompromised patients exhibited hepatic dysfunction, with elevated aspartate aminotransferase (AST) and alanine aminotransferase (ALT) levels. All patients received antiviral therapy with valaciclovir or acyclovir, and some additionally received intravenous immunoglobulin. All five adult patients in this series recovered from varicella without long-term sequelae, although one later died from unrelated causes. We additionally note that one patient later died from cerebral embolism and pneumonia, unrelated to varicella. **Conclusions**: This retrospective case series illustrates the vulnerability of immunocompromised adults to severe varicella and aligns with current recommendations supporting the vaccination of susceptible high-risk adults. While antiviral therapy remains essential for clinical management, prevention through appropriate vaccination strategies—varicella vaccine for preventing primary infection in eligible individuals and RZV for preventing herpes zoster reactivation in immunocompromised adults—represent an important preventive strategy for reducing overall VZV-related morbidity and mortality. These findings reinforce the importance of preventive strategies, including vaccination and early recognition of varicella in high-risk adults.

## 1. Introduction and Clinical Significance

### 1.1. Introduction

Varicella, commonly known as chickenpox, is caused by the varicella-zoster virus (VZV) and is usually a childhood illness, with nearly 90% of cases occurring in children [1]. Although typically mild in pediatric populations, adult varicella carries a 23-to-29-fold higher risk of death and is more likely to cause severe complications such as pneumonia, hepatitis, and encephalitis [2].

Immunocompromised individuals—including those with collagen vascular diseases, hematologic malignancies, or those receiving immunosuppressive therapy—are particularly vulnerable to severe and sometimes life-threatening varicella infections. Previous studies have reported that approximately 70–90% of varicella-related deaths occur in immunocompromised patients, underscoring the critical need for preventive strategies [3].

In Japan, many adults lack documented varicella vaccination, representing a missed opportunity for preventing primary VZV infection. Routine childhood vaccination began in 2014, but adult vaccination is not standard practice. Immunocompromised adults cannot receive the live-attenuated varicella vaccine and therefore remain susceptible to primary infection. For these individuals, recombinant zoster vaccine (RZV) is recommended to prevent VZV reactivation, although it does not prevent primary varicella. Understanding these distinctions is essential for interpreting adult varicella cases.

Despite the recognized severity of adult varicella, real-world clinical data from Japan remain limited. Here, we report five adult cases diagnosed at our institution between 2007 and 2025, who were receiving immunosuppressive therapy or cancer treatment. None of the patients had a known history of varicella vaccination. By describing their clinical courses, we aim to highlight the importance of proactive vaccination strategies and early antiviral therapy in high-risk adult populations.

### 1.2. Clinical Significance

This case series demonstrates the severe clinical course of varicella in immunocompromised adults and underscores the importance of vaccination in preventing life-threatening complications. None had documented evidence of vaccination, revealing a significant gap in preventive care. Strengthening adult immunization strategies and ensuring timely antiviral treatment may reduce morbidity and mortality in high-risk populations.

## 2. Case Presentation

### 2.1. Study Design and Ethical Approval

We conducted a retrospective review of varicella cases diagnosed at Hirosaki University Hospital between April 2007 and March 2025. This study was approved by the Committee of Medical Ethics of Hirosaki University Graduate School of Medicine (Approval No.2020-364).

### 2.2. Case Identification and Diagnostic Criteria

Varicella cases were identified by patients presenting to the Department of Dermatology and reported to the Infection Control Center. All hospitalized cases were captured, although some outpatient cases may have been missed. Diagnosis was made clinically based on the sudden onset of generalized erythematous papules and vesicles at different developmental stages (papules, vesicles, and crusts).

### 2.3. Diagnostic Testing

DermaQuick VZV^®^ is an immunochromatographic assay that detects VZV antigen in vesicular fluid or epithelial cell–containing swab samples. A red-purple test line indicates antigen positivity, with a separate control line confirming assay validity. As this was a retrospective chart review, PCR and viral culture were not performed in any case. The DermaQuick VZV^®^ kit was used as documented in the medical records, and previous studies have demonstrated its high concordance with real-time PCR, supporting its reliability as a rapid diagnostic tool.

### 2.4. Data Collection

Clinical laboratory and treatment data were extracted from medical records. In total, 10 patients were identified, including 2 children and 8 adults (4 males, 4 females), as diagnosed with varicella at Hirosaki University Hospital between April 2007 and March 2025 (the total number of new outpatients during the 18-year period was 184,617). Among the eight adult patients, five provided informed consent and are presented in detail in this report (Table 1).

### 2.5. Case 1

**Figure 1:**
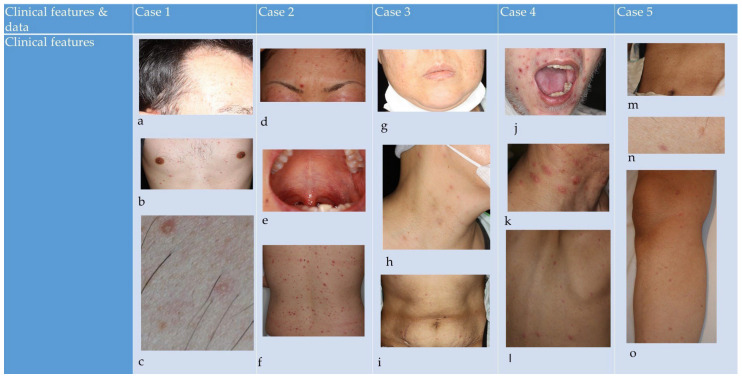
Clinical Presentations of Adult Varicella Cases. (**a**) Case 1: Papulovesicular eruptions on the forehead. (**b**) Case 1: Papulovesicular eruptions on the chest. (**c**) Case 1: Magnified view of the lesions. (**d**) Case 2: Umbilicated vesicles on the forehead. (**e**) Case 2: Oral lesions. (**f**) Case 2: Eroded papules over the lower back. (**g**) Case 3: Vesicles and papules on the face. (**h**) Case 3: Vesicles and papules on the neck. (**i**) Case 3: Vesicles and papules on the abdomen. (**j**) Case 4: Papulovesicular eruptions on the face. (**k**) Case 4: Papulovesicular eruptions on the neck. (**l**) Case 4: Papulovesicular eruptions on the back. (**m**) Case 5: Vesicles on the trunk. (**n**) Case 5: Magnified view of the vesicles. (**o**) Case 5: Additional vesicles on the lesions on one lower limb.

A 37-year-old man with dermatomyositis presented with a 2-day history of epigastric pain, fever, headache, joint pain, and widespread vesicular eruptions (Figure 1a–c). He was receiving prednisolone 30 mg/day and FK506 10 mg/day. He denied prior varicella infection or vaccination. DermaQuick VZV^®^ testing of vesicular fluid was positive. Laboratory tests showed elevated transaminases (AST 53 U/L, ALT 73 U/L), consistent with hepatic involvement. Oral valaciclovir at 3000 mg/day for 7 days led to complete recovery.

### 2.6. Case 2

A 42-year-old woman with systemic lupus erythematosus (SLE), treated with prednisolone 15 mg/day and tacrolimus, developed varicella after exposure to her mother with herpes zoster. She presented with fever and vesicular eruptions affecting the oropharynx and skin (Figure 1d–f). Laboratory tests showed mild liver dysfunction (AST 44 U/L, ALT 49 U/L). She was hospitalized under isolation precautions and treated with intravenous acyclovir for 5 days, followed by oral valaciclovir for 3 days, with improvement over three weeks. Her VZV IgG and IgM levels were as follows: three years before the onset of varicella, the VZV IgG level (ELISA) was 13.9 and the VZV IgM level was 0.37. At the time of varicella onset, the VZV IgG and IgM levels had decreased to 8.6 and 0.22, respectively. Twelve weeks after onset, the VZV IgG level had risen to ≥128 and the VZV IgM level to 0.76.

### 2.7. Case 3

A 44-year-old woman with acute myeloid leukemia (AML) who was receiving hydrocortisone sodium succinate 100 mg/day presented with high fever, fatigue, and widespread vesicular eruptions (Figure 1g–i). Liver function tests showed mildly elevated transaminases (AST 59 U/L, ALT 96 U/L). She was hospitalized under isolation precautions and treated with intravenous immunoglobulin followed by intravenous acyclovir for 10 days, resulting in clinical improvement and discharge on day 14.

Four years later, she died from cerebral embolism and acute pneumonia, events considered unrelated to varicella. No autopsy was performed, preventing differentiation between VZV-associated vasculopathy and thromboembolism. Neuroimaging revealed multiple ischemic lesions in the right cerebral hemisphere, but these findings were insufficient to determine VZV involvement. At the time of death, neither VZV serology nor cerebrospinal fluid (CSF) VZV PCR was obtained, limiting virological assessment. Her relapsed leukemia and ongoing immunosuppression may have increased the risk of thromboembolic events independent of prior VZV infection. These limitations reflect the retrospective nature of the study, which relied solely on available medical records.

### 2.8. Case 4

A 54-year-old man undergoing treatment with pazopanib hydrochloride for left retroperitoneal sarcoma presented with vesicular eruptions on the face and body (Figure 1j–l). A rapid varicella antigen test (DermaQuick^®^) was positive, confirming the diagnosis of varicella. Laboratory findings showed mild liver dysfunction (AST 43 U/L, ALT 40 U/L). He was placed in a private isolation room and received intravenous acyclovir at 750 mg/day for 7 days, resulting in clinical recovery.

### 2.9. Case 5

A patient with recurrent choriocarcinoma was hospitalized in our department and was undergoing MEA chemotherapy (methotrexate, etoposide, actinomycin-D, and leucovorin). On the first day of the third MEA cycle, the patient developed vesicular eruptions on the extremities and was referred to our department. No lesions were observed on the face or trunk; however, several 2–3 mm vesicles were present on both the upper and lower extremities (Figure 1m–o). A rapid varicella antigen test (DermaQuick^®^) was positive, confirming the diagnosis of varicella. Liver function tests revealed moderately elevated transaminases (AST 107 U/L, ALT 131 U/L). She was placed in a private isolation room and received intravenous acyclovir at 750 mg/day for 10 days. Re-epithelialization was achieved by day 16, after which isolation precautions were discontinued.

### 2.10. Use of Generative Artificial Intelligence (GenAI)

Generative artificial intelligence was used solely for language refinement and structural editing of the manuscript text. No GenAI tools were used for data generation, analysis, interpretation, or figure creation. AI-assisted image generation was used to prepare the graphical abstract using ChatGPT (OpenAI), model version GPT-5.5, accessed in June 2026.

## 3. Results

### 3.1. Overview of Cases

Among the eight adult patients identified, five consented to publication and are described in detail.

### 3.2. Case Descriptions

(a) Case 1: A 37-year-old man with papulovesicular eruptions on his head, face, trunk, and limbs.

(b) Case 2: A 42-year-old woman with a rash on her head with umbilicated vesicles on her face, oral lesions, and eroded papules over her trunk and limbs.

(c) Case 3: A 44-year-old woman with vesicles and papules on her head, face, neck, and trunk.

(d) Case 4: A 54-year-old man with papulovesicular eruptions on his head, face, trunk and limbs.

(e) Case 5: A 58-year-old woman with papulovesicular eruptions on her limbs.

### 3.3. Laboratory Findings

All immunocompromised patients demonstrated elevated transaminases, with AST and ALT levels exceeding reference ranges (Table 1).

### 3.4. Treatment and Outcomes

All patients received antiviral therapy; one received IVIG.

All recovered without sequelae.

## 4. Discussion

Adult varicella cases were few in our hospital, but this aligns with national epidemiology in Japan, where surveillance is pediatric-centered and adult primary varicella is rarely captured. As this retrospective case series lacked immunocompetent adult controls, causal inference regarding the impact of immunosuppression cannot be made. However, epidemiological data consistently show that 70–90% of varicella-related deaths occur in immunocompromised individuals [4], and the severe clinical courses observed here are consistent with this known risk profile. Our findings are descriptive and align with current recommendations supporting the vaccination of susceptible high-risk adults.

Varicella vaccination prevents primary VZV infection in immunocompetent individuals, whereas immunocompromised adults cannot receive the live-attenuated vaccine and remain susceptible. One patient in our series acquired varicella after household exposure to herpes zoster, illustrating that reactivated VZV can transmit to susceptible adults. Recombinant zoster vaccine (RZV), which prevents VZV reactivation, is recommended for immunocompromised adults and, in some guidelines, for their household contacts.

Severe complications such as hepatic dysfunction, acute liver failure, and thrombocytopenia have been reported among immunocompromised patients [5,6,7,8]. Early acyclovir therapy reduces viremia and remains essential [9]. Varicella dissemination in immunocompromised hosts reflects impaired cellular immunity, causing predictable multi-organ involvement [10]. Comparative pathogenesis studies demonstrate that severity correlates with delayed antiviral responses [10]. The uniform hepatic dysfunction in all five patients confirms VZV dissemination as an expected complication requiring standardized surveillance and early intervention. The degree of transaminase elevation appeared to reflect the intensity of immunosuppression.

Despite strong immunogenicity and long-term protection of varicella vaccination [11], adult varicella vaccination documentation gaps persist globally despite varied healthcare infrastructure. A 2024 New York City outbreak investigation identified 873 cases, with 92% having no documentation of varicella vaccination [12], substantially exceeding Japan’s 2014–2021 data showing that 26% lack documentation and 70% unaware of status. Recent surveillance conducted in 2025 in Florida reports that the majority of adults aged 19+ with varicella lacked adequate vaccination documentation or had unknown status, with 53% being unvaccinated [13]. Simultaneously, surveillance in Ningbo, China (2010–2023) documented an epidemiological shift toward older age groups despite exceeding 90% first-dose vaccination coverage, suggesting systematic adult immunization gaps across Asia-Pacific and North American regions [14]. Reports demonstrate that many adults lack documented immunity and contact with herpes zoster is a common source of exposure. In our series, none of the patients had documented vaccination. Case 2 demonstrates secondary transmission: the patient contracted varicella following household exposure to herpes zoster in a family member, illustrating the risk of VZV transmission from reactivated virus in immunocompromised hosts.

Immunocompromised adults are at increased risk for both severe varicella and herpes zoster reactivation. Vaccination of these individuals—and when appropriate, their close contacts—is therefore important to reduce VZV-related disease burden. Because VZV establishes lifelong latency, herpes zoster represents reactivation rather than new infection, yet vesicular fluid can transmit VZV to susceptible individuals, underscoring the ongoing public health relevance of VZV.

Varicella vaccine prevents primary infection, whereas recombinant zoster vaccine (RZV) prevents herpes zoster reactivation and does not prevent primary varicella. Overall, our findings highlight the vulnerability of immunocompromised adults to severe varicella, persistent gaps in adult vaccination coverage, and the value of rapid antigen testing and standardized monitoring of hepatic involvement. International guidelines from the CDC, NACI, and WHO recommend RZV for immunocompromised adults [15,16,17], ideally before initiating immunosuppressive therapy or during periods of stable disease. Strengthening adult immunization strategies—varicella vaccine for preventing primary infection in eligible individuals and RZV for preventing herpes zoster reactivation—represents an important public health measure to reduce VZV-related morbidity and mortality.

## 5. Conclusions

The findings are consistent with current recommendations supporting the vaccination of susceptible high-risk adults. This case series illustrates the vulnerability of immunocompromised adults to severe varicella and highlights persistent gaps in vaccination coverage. While antiviral therapy remains essential for clinical management, prevention through appropriate vaccination is central to reducing disease burden. From the broader perspective of physical, mental, and social well-being, protecting high-risk adults and their household contacts through prioritized herpes zoster vaccination is essential. Strengthening adult immunization strategies will contribute to improved long-term health outcomes in immunocompromised populations.

## 6. Limitations

This study was limited by its retrospective single-center design and small sample size. In addition, no immunocompetent control group was included, preventing causal inference regarding the impact of immunosuppression on disease severity. Vaccination histories were not available for all patients, and some mild outpatient cases may have been missed.

## Figures and Tables

**Table 1:** Clinical and Laboratory Characteristics of Adult Varicella Cases.

Case	Case 1	Case 2	Case 3	Case 4	Case 5
Age (years)	37	42	44	54	58
Sex	Male	Female	Female	Male	Female
WBC count (/µL)	11,240	5940	3090	2540	4560
Neutrophil count (/µL)	9100	2540	1330	2130	3970
Lymphocyte count (/µL)	900	700	460	180	510
AST (U/L)	53	44	59	43	107
ALT (U/L)	73	49	96	40	131
γ-GTP (U/L)	26	66	80	79	241
T-Bil (mg/dL)	0.7	0.4	0.4	0.5	0.6
LDH (U/L)	318	309	188	248	259
CRP (mg/dL)	0.7	0.63	0.98	6.62	0.81
Varicella vaccination history	No	No	Uncertain	Uncertain	Uncertain
Past medical history	Dermatomyositis	SLE	AML	left retroperitoneal sarcoma	choriocarcinoma
Corticosteroid treatment	Prednisolone 30 mg/day	Prednisolone 15 mg/day	Hydrocortisone 100 mg/day	pazopanib hydrochloride	Methotrexate, etoposide, dactinomycin
Antiviral agents	Valaciclovir hydrochloride was administered orally at 3000 mg per day	Acyclovir at 1500 mg per day for 5 days → Valaciclovir 3000 mg/day × 3 days	Acyclovir at 250 mg per day for 10 days.	Acyclovir at 750 mg per day for 7 days.	Acyclovir at 750 mg per day for 10 days.
Administration	−	+	+	+	+
Hospitalization duration	0	5	14	8	16
Time of initiation	14 days	21 days	14 days	8 days	16 days
IVIG	−	−	+	−	−

ALT: Alanine aminotransferase (reference range: 10–42 U/L); AML: Acute myeloid leukemia; AST: Aspartate aminotransferase (reference range: 13–30 U/L); CRP: C-reactive protein (reference range: 0.00–0.14 mg/dL); LDH: Lactate dehydrogenase, measured using the International Federation of Clinical Chemistry and Laboratory Medicine (IFCC) method (reference range: 124–222 U/L); SLE: Systemic lupus erythematosus; T-Bil: Total bilirubin (reference range: 0.4–1.5 mg/dL); WBC: White blood cell count (reference range: 3300–8600/µL); γ-GTP: Gamma-glutamyl transferase (reference range: 13–64 U/L). Varicella vaccination history was categorized based on medical records or patient recall. Corticosteroid treatment indicates the type and daily dosage at the onset of varicella. Administration: “−” indicates outpatient management only, and “+” indicates that inpatient treatment was required.

## Data Availability

The data presented in this study are available on request from the corresponding author due to privacy and ethical restrictions.

## Abbreviations

The following abbreviations are used in this manuscript:

VZV	Varicella-zoster virus
HZ	Herpes zoster
PCR	Polymerase chain reaction
CT	Computed tomography
IVIG	Intravenous immunoglobulin
AST	Aspartate aminotransferase
ALT	Alanine aminotransferase
LDH	Lactate dehydrogenase
CRP	C-reactive protein
WBC	White blood cell count
IgG/IgM	Immunoglobulin G/M
MMF	Mycophenolate mofetil
PSL	Prednisolone
CyA	Cyclosporine A
